# Synthesis of an aza[5]helicene-incorporated macrocyclic heteroarene via oxidation of an *o*-phenylene-pyrrole-thiophene icosamer

**DOI:** 10.3762/bjoc.21.119

**Published:** 2025-07-31

**Authors:** Yusuke Matsuo, Aoi Nakagawa, Shu Seki, Takayuki Tanaka

**Affiliations:** 1 Department of Molecular Engineering, Graduate School of Engineering, Kyoto University, Kyotodaigakukatsura, Nishikyo-ku, Kyoto, Japan; 2 Department of Chemistry, Graduate School of Science, Kyoto University, Kitashirakawa Oiwake-cho, Sakyo-ku, Kyoto, Japan

**Keywords:** cyclophane, fluorescence, heterohelicene, intramolecular oxidative coupling

## Abstract

The intramolecular oxidative fusion reaction of macrocyclic heteroaromatic arrays has provided strained polycyclic heteroaromatic macrocycles as promising functional molecules. In this study, we prepared an *ortho*-phenylene-pyrrole-thiophene hybrid icosamer, as the largest cyclic array in the series. The oxidative fusion reaction with [bis(trifluoroacetoxy)iodo]benzene (PIFA) afforded a cyclophane-type aza[5]helicene-incorporated macrocycle, the structure of which was unambiguously revealed by X-ray diffraction analysis. Its optical properties have been investigated in detail.

## Introduction

**Figure 1:**
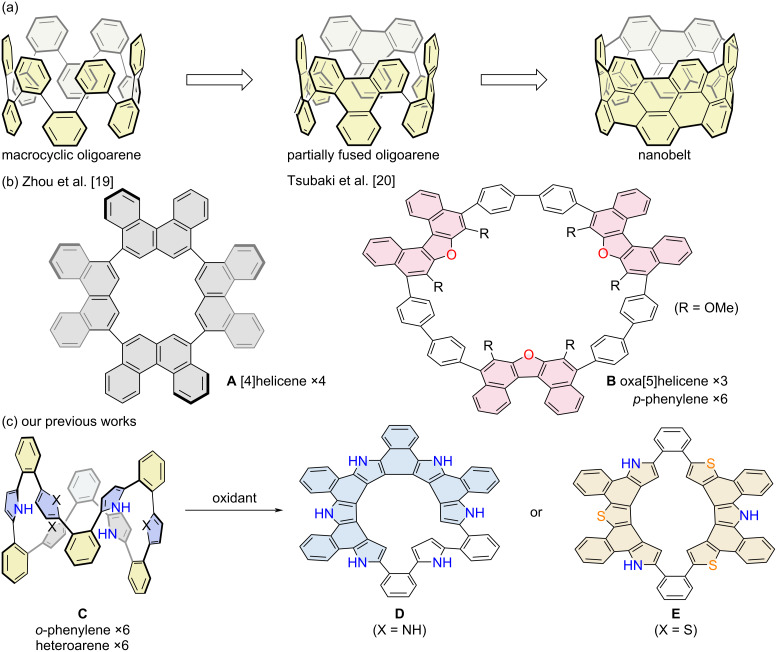
(a) Increased ring-strain from macrocyclic oligoarene to partially fused oligoarene and nanobelt. (b) Cyclo[4]helicenylene and cyclo(oxa[5]helicenylene-biphenylene). (c) Intramolecular oxidative coupling of cyclic *o*-phenylene-pyrrole-thiophene dodecamers.

Conjugated macrocyclic polyarenes have attracted significant attention due to their stimuli-responsive optoelectronic properties, dynamic structural changes, and host–guest interactions [1–5]. In addition to these promising functionalities, their cyclic polyaromatic frameworks can be further transformed into fused structures. To this end, belt-like polyaromatic architectures can be developed, inspiring ongoing efforts toward the construction of carbon nanotube analogs (Figure 1) [6–12]. Nevertheless, partially fused macrocyclic intermediates are also important as they exhibit structural strain associated with both the polycyclic segments and the inherent strain stemming from the macrocyclic structure. For instance, cyclic chrysenylenes [13–16] and pyrenylenes [17–18] were reported to adopt unique chiral arrangements depending on their stereochemistry. Helical motifs such as carbo[4]helicene and oxa[5]helicene were incorporated into cyclic structures, giving rise to cyclic carbo[4]helicenylene **A** and cyclic oxa[5]helicenylene-biphenylene **B**, respectively [19–20]. Recently, our group established an efficient synthetic strategy for strained macrocyclic polyarenes, such as compound **C**, in which *o*-phenylene units preorganize adjacent heteroaromatics into close proximity, thereby facilitating oxidative ring-closure reactions [21]. Among these, the largest macrocycle ever synthesized is a dodecameric hybrid array of 1,2-phenylene, 2,5-thienylene, and 2,5-pyrrolylene units [22–23]. The intramolecular oxidative coupling of these arrays afforded heterohelicene-incorporated macrocycles **D** and **E**, depending on the relative arrangements of the pyrrole and thiophene units [24–25]. The influence of heteroaromatic positioning on the reaction outcome has been rationalized in our previous work [25]. As a further extension of this molecular design, herein we report the synthesis of an *o*-phenylene-pyrrole-thiophene hybrid icosamer and its oxidative fusion to yield an aza[5]helicene-incorporated macrocycle. The resulting cyclophane-like structure and its optical properties have been analyzed in detail.

## Results and Discussion

### Synthesis and characterization

#### Synthesis

**Scheme 1:**
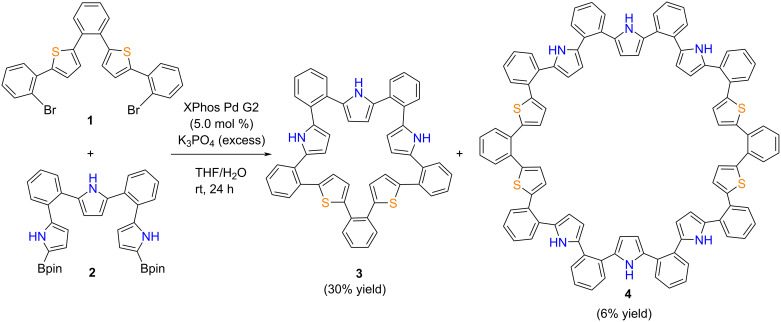
Synthesis of *o*-phenylene-pyrrole-thiophene hybrid macrocycles.

We obtained *o*-phenylene-pyrrole-thiophene hybrid icosamer **4** during our attempt to synthesize hybrid decamer **3** in a previous report [26], via a Suzuki–Miyaura cross-coupling reaction between dibromo precursor **1** and borylated precursor **2** (Scheme 1). The resulting mixture was successfully separated by column chromatography on silica using CH_2_Cl_2_/*n*-hexane as an eluent to give icosamer **4** in 6% yield, along with decamer **3** (30%). High-resolution atmospheric-pressure-chemical-ionization time-of-flight mass-spectrometry (HR-APCI-TOF-MS) showed a molecular ion peak for **4** at *m/z* = 1479.4320 (calcd for C_100_H_66_N_6_S_4_, *m/z* = 1479.4305). The ^1^H NMR spectrum of **4** in acetone-*d*_6_ exhibited two NH signals at 9.07 and 8.98 ppm and five doublet signals due to the heterole β-protons in the range of 6.7–5.8 ppm, along with *o*-phenylene protons around 7 ppm.

**Figure 2:**
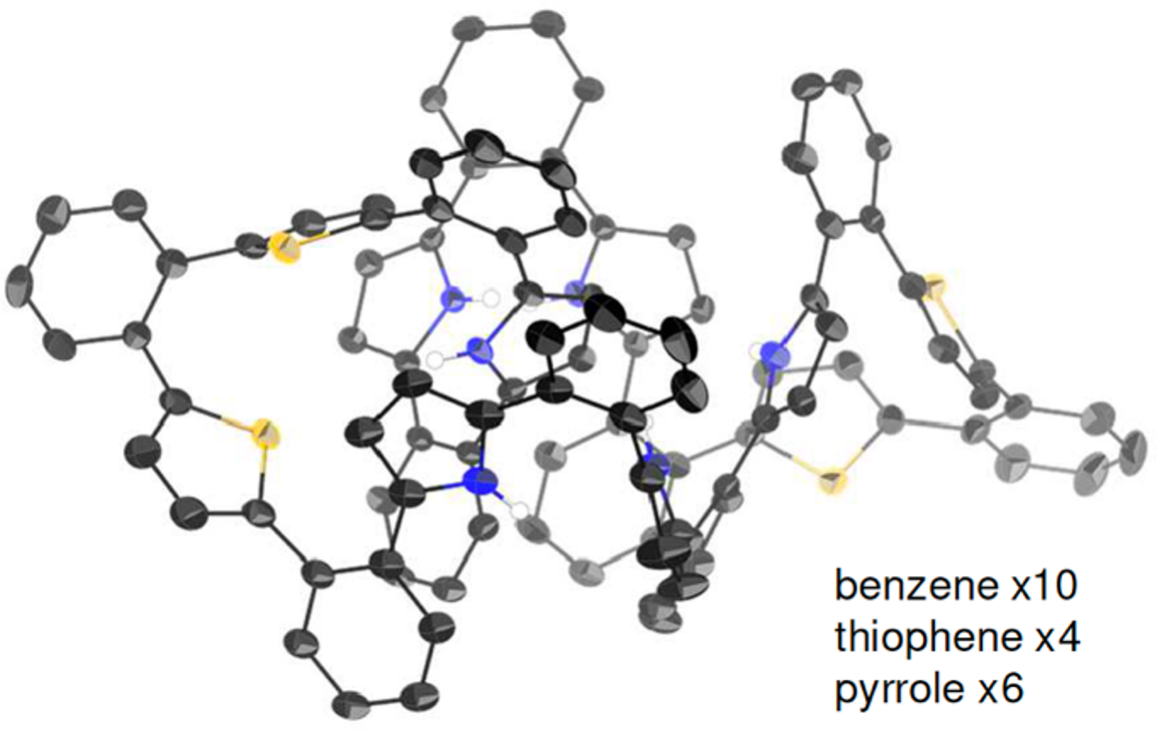
X-ray crystal structure of **4**. Thermal ellipsoids are scaled to 50% probability level. Solvent molecules and hydrogen atoms except for NHs are omitted for clarity.

Single crystals suitable for X-ray diffraction analysis were obtained from a mixture of acetone/*n*-hexane and the solid-state structure was successfully determined (Figure 2). Similar to other previously reported *o*-phenylene-bridged hybrid nanorings [22–23], the average dihedral angles were 40.66° between the phenylene and pyrrole units, and 57.22° between the phenylene and thiophene units. This represents the largest *o*-phenylene-bridged heteroaromatic macrocycle whose structure has been confirmed by X-ray diffraction analysis.

**Scheme 2:**
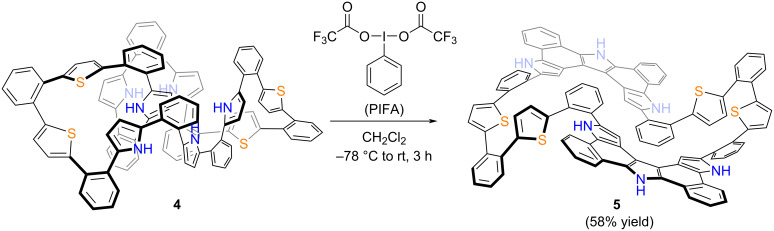
Synthesis of aza[5]helicene-incorporated macrocyclic heteroarene **5**.

**Figure 3:**
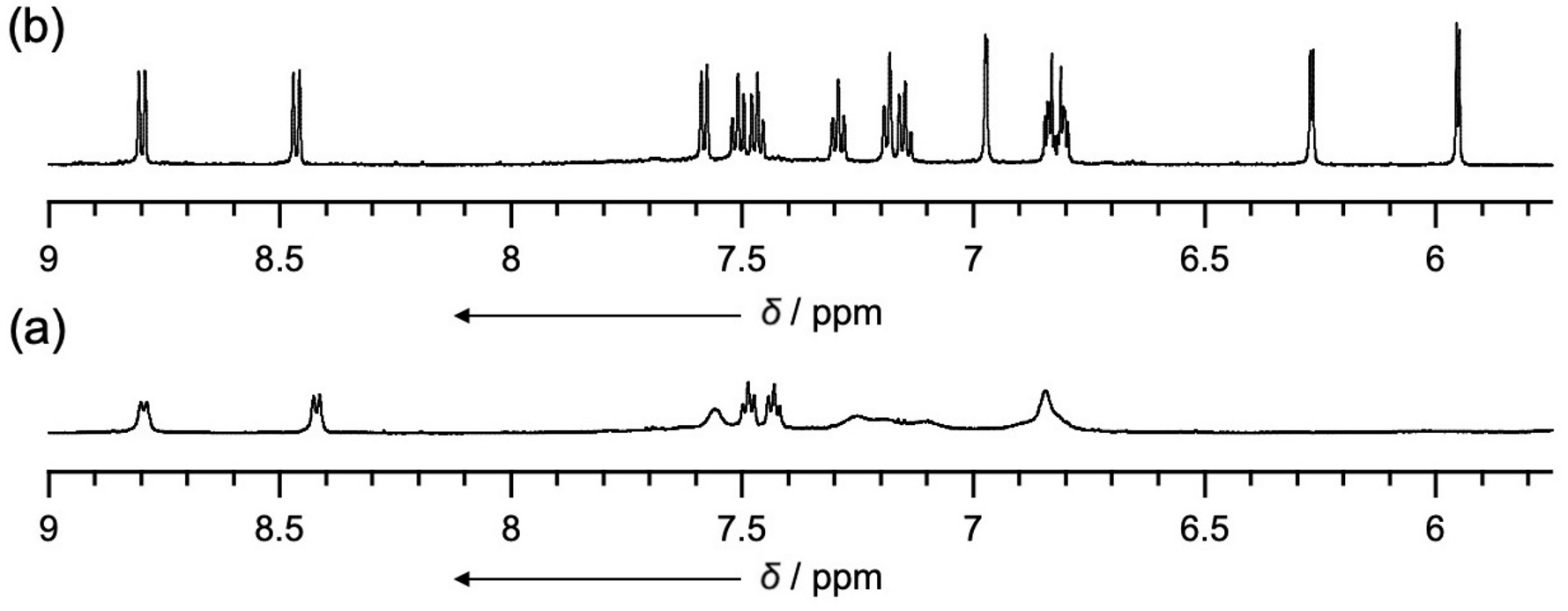
^1^H NMR spectra of **5** in DMSO-*d*_6_ (a) at room temperature and (b) at 100 °C.

**Figure 4:**
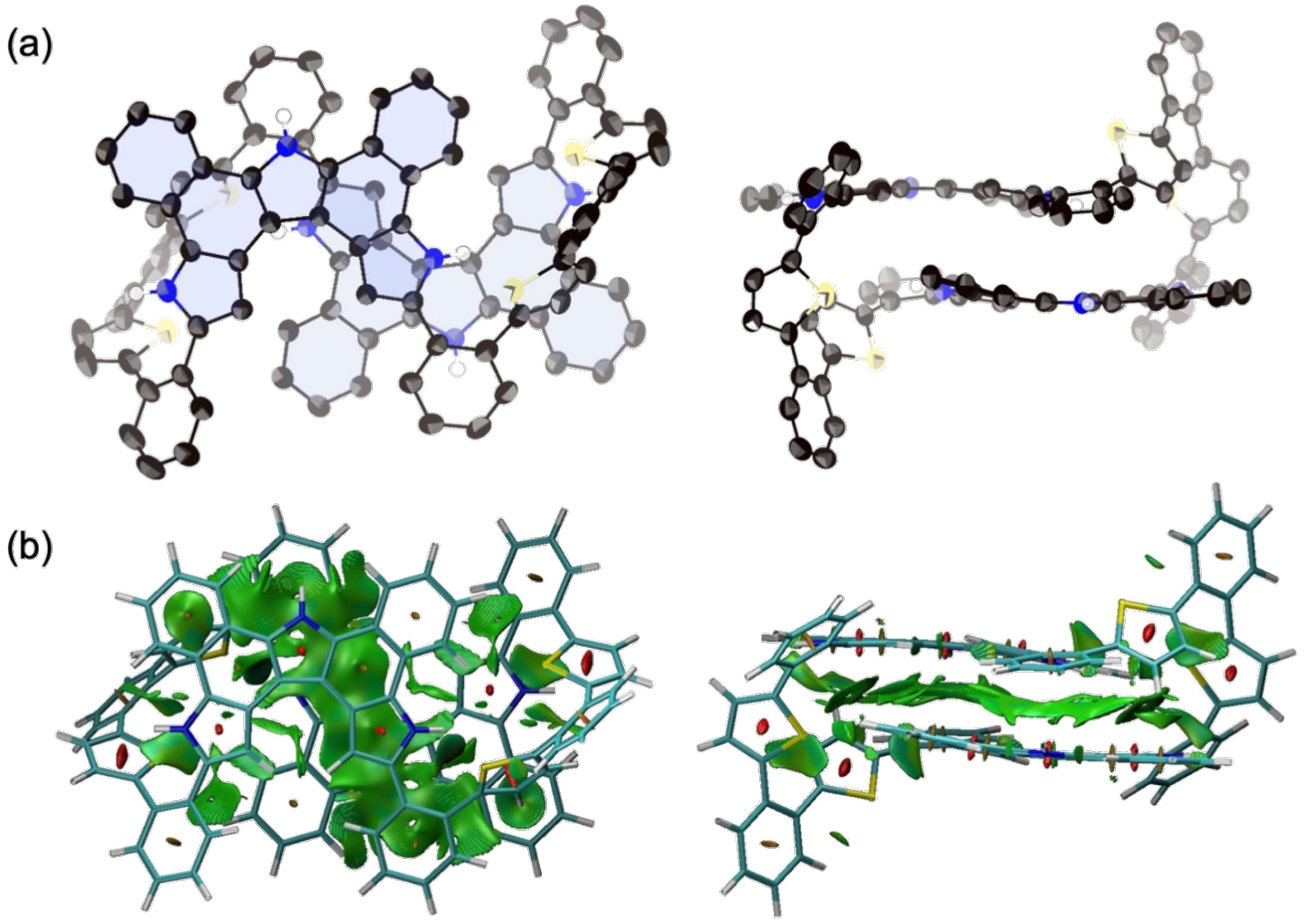
(a) X-ray crystal structure of **5**; (left) top view, (right) side view. Thermal ellipsoids are scaled to 50% probability level. Solvent molecules and hydrogen atoms except for NHs are omitted for clarity. (b) NCI plot of **5**; (left) top view, (right) side view (isosurface: 0.50, range: −0.03 < sign(λ_2_)ρ < 0.03).

Next, oxidation of **4** was attempted using [bis(trifluoroacetoxy)iodo]benzene (PIFA) in CH_2_Cl_2_ at −78 °C (Scheme 2). These reaction conditions had previously proven effective for the oxidation of **3** and other *o*-phenylene-bridged acyclic heteroaromatics [26–27]. Thus, to a solution of **4** in CH_2_Cl_2_ was added 15 equivalents of PIFA at −78 °C and stirred for 3 h. The mixture was then allowed to warm to room temperature to give a dark solution. The system was worked-up with NaBH_4_/MeOH for 10 minutes followed by extraction with CH_2_Cl_2_ and evaporation of the solvent to afford a crude product, which was recrystallized from THF to give **5** in 58% yield. Due to its poor solubility in common organic solvents, the ^1^H NMR spectrum could only be recorded in DMSO. At room temperature, the ^1^H NMR spectrum in DMSO-*d*_6_ exhibited broad signals in the range of 6–7 ppm, which sharpened significantly at 100 °C (Figure 3). The ^1^H NMR spectrum at 100 °C displayed distinct signals at 12.01 and 11.54 ppm due to NH protons, a singlet for the pyrrole β-protons at 6.97 ppm, and doublets for the thiophene β-protons at 6.26 and 5.95 ppm. HR-APCI-TOF-MS revealed a molecular ion peak at *m/z* = 1471.3682 (calcd for C_100_H_58_N_6_S_4_, *m/z* = 1471.3679), indicating the loss of eight hydrogen atoms from **4**, suggesting the formation of a fused structure at the pyrrole segments. Finally, the structure was unambiguously revealed by X-ray diffraction analysis to display an aza[5]helicene-incorporated macrocyclic structure (Figure 4). In the solid-state, the distance between the two aza[5]helicene moieties was found to be 3.185 Å, closely consistent with the DFT-optimized value of 3.136 Å (see Supporting Information File 1). The average dihedral angles between the *o*-phenylene and aza[5]helicene segments, and between the *o*-phenylene and thiophene segments, were 37.52° and 44.28°, respectively. Four NH sites of the aza[5]helicene moiety formed hydrogen bonds with DMSO molecules in the crystal lattice, as observed in aza[*n*]helicenes recently reported [28], while the other two NH sites remained uncoordinated due to steric hindrance. Non-covalent interaction (NCI) plot analysis revealed distinct intramolecular π–π dispersion interactions between the two aza[5]helicene moieties (green surface in Figure 4b) [29–31].

#### Optical properties

**Figure 5:**
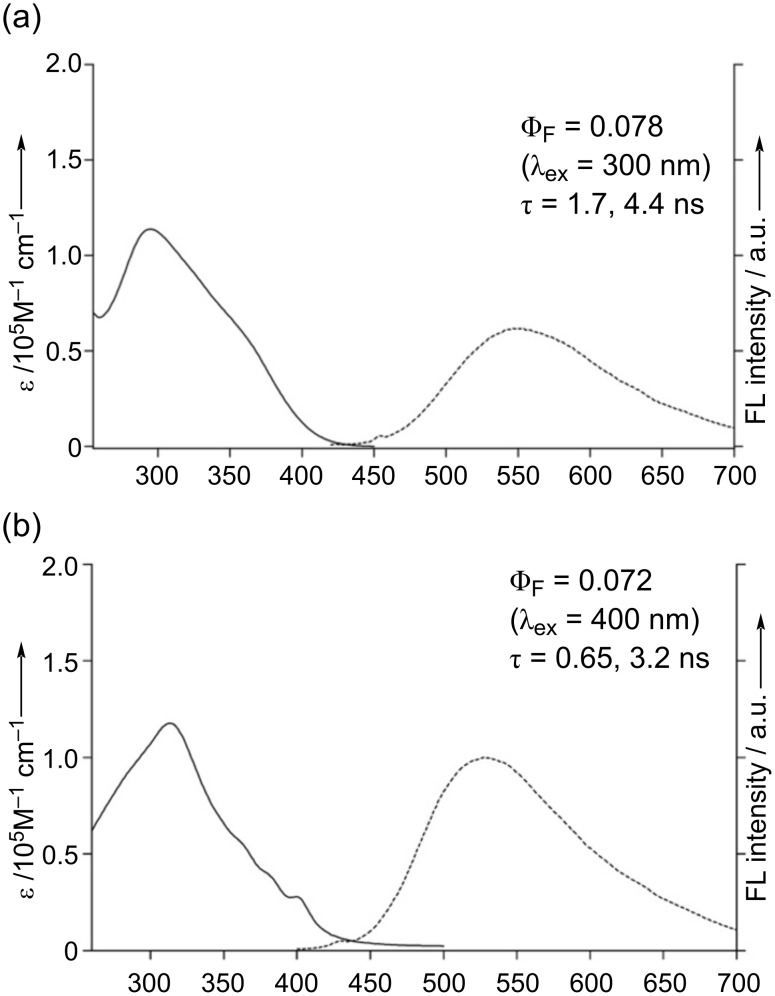
UV–vis absorption and emission spectra of (a) **4** and (b) **5** in DMSO.

The electronic absorption and emission spectra of **4** were measured in DMSO (Figure 5a). As observed for other *o*-phenylene-bridged cyclic heteroarenes in previous reports, compound **4** exhibited a broad featureless absorption band up to 450 nm, with emission peaked at 546 nm. The red-shifted emission is likely due to a significant structural relaxation in the excited state. The fluorescence quantum yield (Φ_F_) was determined as 0.078 (λ_ex_ = 300 nm), and the fluorescence lifetime (τ) using biexponential decay model fitting as 1.7 and 4.4 ns. The partially fused structure of **5** exhibited a well-defined lowest-energy absorption band peaked at 399 nm (Figure 5b). A broad emission was observed at 528 nm, resulting in a relatively large Stokes shift of 6100 cm^−1^, which can be attributed to the structural relaxation in the excited state, as inferred by the observed broad ^1^H NMR spectrum at room temperature. Due to the thermal energy loss, the Φ_F_ value was modest (0.072), which is lower than those of related aza[*n*]helicene analogs [27–28]. The fluorescence lifetime (τ) was determined by biexponential decay model fitting as 0.65 and 3.2 ns. DFT calculation was conducted to investigate the electronic structure. The HOMO and HOMO−1 are primarily localized on the aza[5]helicene moieties, while the orbital coefficients are distributed to the bridging thiophene and *o*-phenylene units in LUMO, indicating a charge-transfer (CT)-like transition. However, further optical characterization of compound **5** was limited due to its poor solubility in common organic solvents.

## Conclusion

A novel *o*-phenylene-pyrrole-thiophene hybrid macrocycle (icosamer **4**) was synthesized via Suzuki–Miyaura cross-coupling and isolated in 6% yield. Oxidation of **4** with PIFA produced a partially fused aza[5]helicene-containing macrocycle **5** in 58% yield, which was also characterized by X-ray analysis and NMR spectroscopy at elevated temperatures. Optical studies showed that compound **4** had broad absorption (up to 450 nm) and emission at 546 nm, while macrocycle **5** showed an emission peak at 528 nm, presumably as a consequence of structural relaxation and CT character. This study illuminated that a partially fused macrocyclic molecule is an intriguing structural motif which comprises a rigid backbone, yet showing somewhat flexible structural dynamics under ambient temperature conditions.

## Supporting Information

File 1 Experimental procedures, characterization data of all products, copies of ^1^H and ^13^C NMR spectra, optical data, and DFT calculation results.

File 2 Crystallographic Information File for compound **4**.

File 3 Crystallographic Information File for compound **5**.

## Data Availability

All data that supports the findings of this study is available in the published article and/or the supporting information of this article.
