# Supplementary material for: Synthesis of an aza[5]helicene-incorporated macrocyclic heteroarene via oxidation of an o-phenylene-pyrrole-thiophene icosamer
Source: Beilstein J Org Chem. 2025 Jul 31;21:1561–7. doi: 10.3762/bjoc.21.119 (PMC12320735; doi:10.3762/bjoc.21.119)
Supplement: File 1 — Experimental procedures, characterization data of all products, copies of 1H and 13C NMR spectra, optical data, and DFT calculation results. [file Beilstein_J_Org_Chem-21-1561-s001.pdf]

BEILSTEIN JOURNAL OF ORGANIC CHEMISTRY

Supporting Information

for

Synthesis of an aza[5]helicene-incorporated macrocyclic heteroarene via oxidation of an *o*-phenylene-pyrrole-thiophene icosamer

Yusuke Matsuo, Aoi Nakagawa, Shu Seki and Takayuki Tanaka

Beilstein J. Org. Chem. **2025,** *21,* 1561–1567. doi:10.3762/bjoc.21.119

Experimental procedures, characterization data of all products, copies of ^{1}H and ^{13}C NMR spectra, optical data, and DFT calculation results

License and Terms: This is a supporting information file under the terms of the Creative Commons Attribution License (https://creativecommons.org/licenses/by/4.0). Please note that the reuse, redistribution and reproduction in particular requires that the author(s) and source are credited and that individual graphics may be subject to special legal provisions.
The license is subject to the *Beilstein Journal of Organic Chemistry* terms and conditions: (https://www.beilstein-journals.org/bjoc/terms)

Table of contents

1. Instrumentation and materials	S2
2. Experimental section	S3
3. NMR spectra	S5
4. Mass spectra	S8
5. X-ray crystallographic details	S9
6. Absorption and fluorescence spectra	S11
7. DFT calculations	S12
8. References	S16

1. Instrumentation and materials

Synthesis

Commercially available solvents and reagents were used without further purification unless otherwise noted. 1,2-Bis(5-bromothiophen-2-yl)benzene,[S1] 2,5-bis(2-(1*H*-pyrrol-2-yl)phenyl)thiophene,[S1] and 2,5-bis(2-(5-(4,4,5,5-tetramethyl-1,3,2-dioxaborolan-2-yl)-1*H*-pyrrol-2-yl)phenyl)-1*H*-pyrrole[S2] were prepared according to the reported procedures. Dry toluene and dichloromethane were used after distillation. Dry THF and dioxane were obtained by passing through alumina under N_2 in a solvent purification system. The spectroscopic grade solvents were used as solvents for all spectroscopic studies. Silica gel column chromatography was performed on Wako gel C-400. Thin-layer chromatography (TLC) was carried out on aluminum sheets coated with silica gel 60 F_{254} (Merck 5554).

NMR and mass spectra

^{1}H and ^{13}C NMR spectra were recorded on a JEOL ECA-600 spectrometer (operating as 600 MHz for ^{1}H and 151 MHz for ^{13}C) and chemical shifts were reported as the δ scale in ppm relative to internal standards $CHCl_3$ (δ = 7.26 ppm for ^{1}H, 77.16 ppm for ^{13}C), DMSO (δ = 2.50 ppm for ^{1}H, 39.52 ppm for ^{13}C), and acetone (δ = 2.05 ppm for ^{1}H, δ = 29.84 ppm for ^{13}C). HR-APCI-TOF-MS was recorded on a BRUKER micrOTOF model or Thermo Fisher Scientific LTQ orbitrap XL model using positive mode.

Optical properties

UV–vis absorption spectra were recorded on a Shimadzu UV-3600. Fluorescence spectra were recorded on a JASCO FP-8500 spectrometer. Absolute fluorescence quantum yields were determined on a HAMAMATSU C9920-02S. Fluorescence lifetime was recorded on a Hamamatsu Photonics Quantaurus-Tau C11367.

X-ray crystallography

Single-crystal diffraction analysis data were collected at −180 °C with a Rigaku XtaLAB P200 by using graphite monochromated Cu *K*α radiation (λ = 1.54187 Å) or with a Rigaku Saturn724+ CCD diffractometer with a graphite-monochromated Mo *K*α radiation (λ = 0.71073 Å). The structures were solved by direct methods (SHELXT-2014/5) and refined with full-matrix least squares technique (SHELXL-2014/7).[S3]

2. Experimental section

Synthesis of compound 4 (along with compound 3)

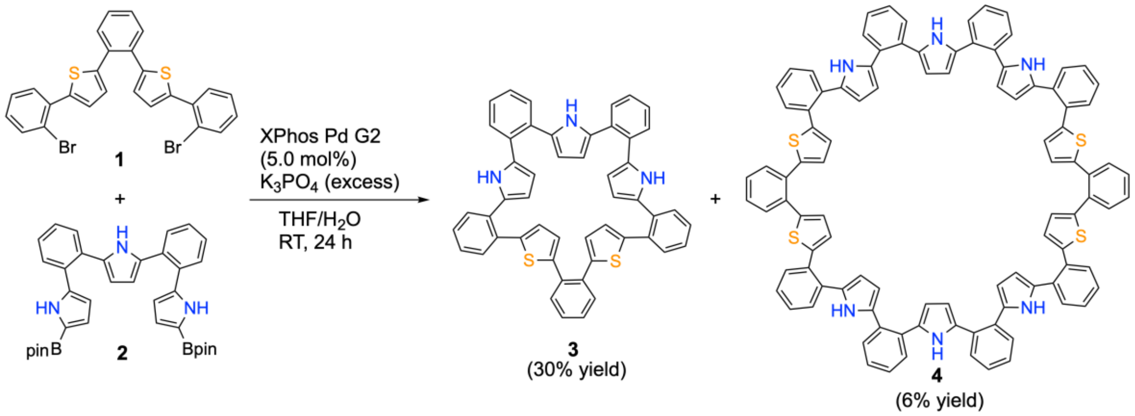

Procedure (in a manner similar to ref. S4): A 2 L three-necked round-bottomed flask was charged with XPhos Pd G2 (365 mg, 0.47 mmol, 5.0 mol %), **1** (5.12 g, 9.3 mmol), and **2** (6.13 g, 10.2 mmol, 1.1 equiv). The reaction flask was evacuated and purged with argon three times. After the mixture was dissolved in dry THF (720 mL) and 0.5 M tripotassium phosphate solution (200 mL, 100 mmol, 11 equiv), the reaction mixture was stirred at room temperature for 24 h. After quenching with saturated aqueous ammonium chloride solution, the mixture was extracted with dichloromethane three times and the combined organic layers were washed with water and brine, and dried over anhydrous sodium sulfate. After removal of the solvents in vacuo, the residue was purified by column chromatography on silica with dichloromethane/*n*-hexane (*v/v* = 1/3) as eluent gave **4** (396 mg, yield: 6%) as pale yellow solids. **m.p.** 186.5-203.0 °C. **^{1}H NMR** (600 MHz, acetone-d_6, room temperature) δ = 9.07 (s, 4H, pyrrole), 8.98 (s, 2H, pyrrole), 7.36–7.35 (m, 4H, phenyl), 7.30–7.29 (m, 12H, phenyl), 7.24–7.16 (m, 16H, phenyl), 7.06–7.03 (m, 8H, phenyl), 6.65 (d, J = 3.7 Hz, 4H, thiophene), 6.57 (d, J = 3.7 Hz, 4H, thiophene), 5.99 (d, J = 2.3 Hz, 4H, pyrrole), 5.95 (dd, J = 6.0, 3.0 Hz, 4H, pyrrole), and 5.87 (dd, J = 6.0, 3.0 Hz, 4H, pyrrole) ppm. **^{13}C NMR** (101 MHz, acetone-d_6, room temperature) δ = 144.52, 143.38, 134.23, 133.22, 132.73, 132.58, 132.44, 131.89, 131.80, 131.69, 131.55, 131.54, 130.18, 130.01, 129.78, 128.95, 128.92, 128.20, 127.74, 127.59, 127.53, 127.41, 110.88, 110.02, and 109.91 ppm. **HR-APCI-TOF-MS** Found: m/z = 1479.4320 $[M]^+$, calcd for $C_{100}H_{66}N_6S_4$: m/z = 1479.4305. **UV/vis** (DMSO, room temperature): λ_{max} / nm (ε / M^{-1} cm^{-1}) = 295 (1.1×10^5). **Fluorescence** (DMSO, λ_{ex} = 300 nm) λ_{max} = 551 nm (Φ_F = 0.078).

Synthesis of compound 5

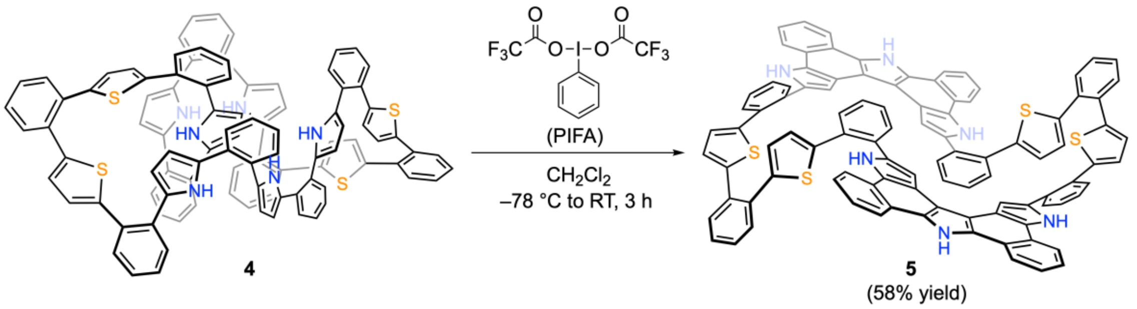

Procedure (in a manner similar to Ref S5): A 200 mL three-necked round-bottomed flask was charged with **4** (74.0 mg, 0.050 mmol) and dry dichloromethane (120 mL) under argon, which was cooled to −78 °C. PIFA (323 mg, 0.75 mmol, 15 equiv) was then added, and the reaction mixture was kept at −78 °C for 3 h. Then, the reaction mixture was allowed to warm to ambient temperature. Next, excess amount of sodium borohydride and methanol were added, and the reaction was kept for 10 min. The mixture was extracted with dichloromethane three times and the combined organic layers were washed with water and brine, and dried over anhydrous sodium sulfate. After removal of the solvents in vacuo, recrystallization from THF gave **5** (42.9 mg, yield: 58%) as pale yellow solid. **m.p.** >350 °C (decomp.). **^{1}H NMR** (600 MHz, DMSO-d_6, 100 °C) δ = 12.02 (s, 2H, pyrrole), 11.54 (s, 4H, pyrrole), 8.81 (d, J = 7.8 Hz, 4H, phenyl), 8.47 (d, J = 7.8 Hz, 4H, phenyl), 7.59 (d, J = 7.8 Hz, 4H, phenyl), 7.52 (t, J = 7.6 Hz, 4H, phenyl), 7.47 (t, J = 7.3 Hz, 4H, phenyl), 7.30 (t, J = 7.3 Hz, 4H, phenyl), 7.19 (d, J = 7.3 Hz, 4H phenyl), 7.15 (t, J = 7.6 Hz, 4H, phenyl), 6.97 (d, J = 2.4 Hz, 4H, pyrrole), 6.84–6.82 (m, 8H, phenyl), 6.26 (d, J = 3.7 Hz, 4H, thiophene), and 5.94 (d, J = 3.7 Hz, 4H, thiophene) ppm. **^{13}C NMR** (151 MHz, DMSO-d_6, 100°C) δ = 142.31, 141.04, 133.53, 132.89, 132.20, 131.91, 130.30, 129.38, 129.36, 128.80, 126.85, 126.79, 126.71, 126.50, 126.20, 125.83, 123.38, 122.47, 121.78, 120.90, 120.54, 119.47, 118.45, 112.78, and 104.35 ppm. **HR-APCI-TOF-MS** Found: m/z = 1471.3682 $[M]^+$, calcd for $C_{100}H_{58}N_6S_4$: m/z = 1471.3679. **UV/vis** (THF, room temperature): λ_{max} / nm (ε / M^{-1} cm^{-1}) = 313 (1.2×10^5) and 399 (2.8×10^4). **Fluorescence** (THF, λ_{ex} = 400 nm) λ_{max} = 528 nm (Φ_F = 0.072).

3. NMR spectra

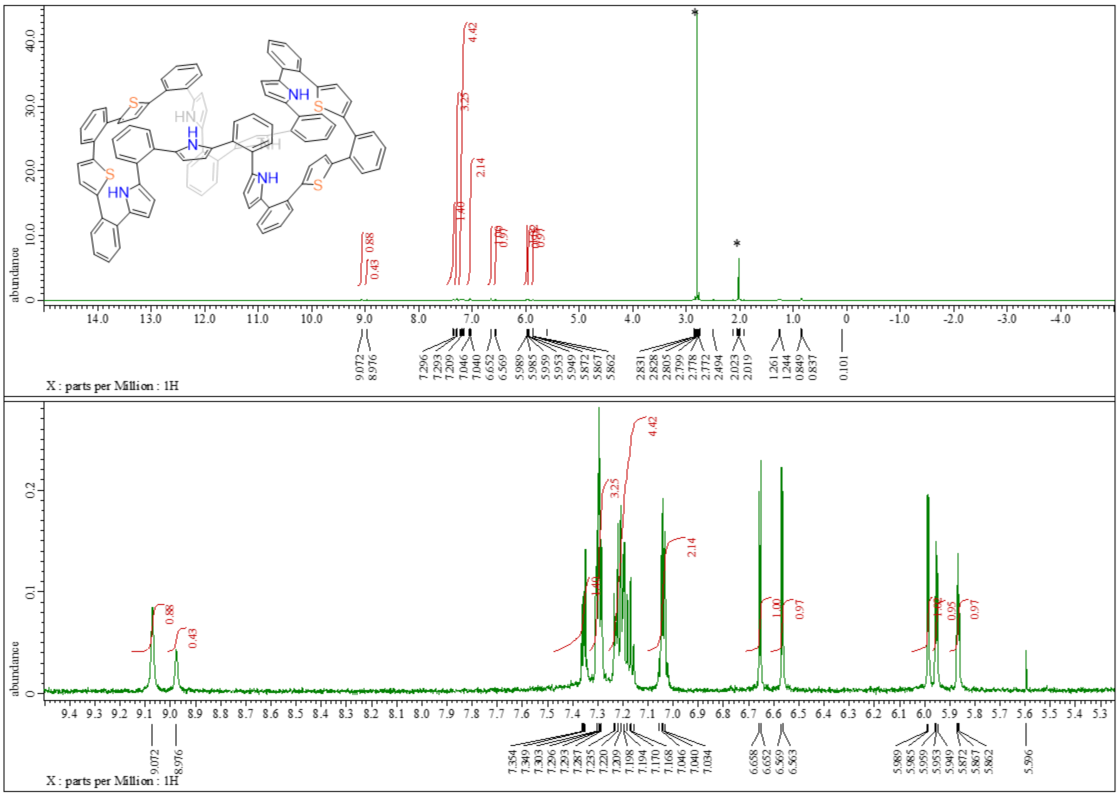

Figure S1. ^{1}H NMR spectrum of **4** in acetone-d_6 at room temperature. Peaks with * are due to the residual solvents.

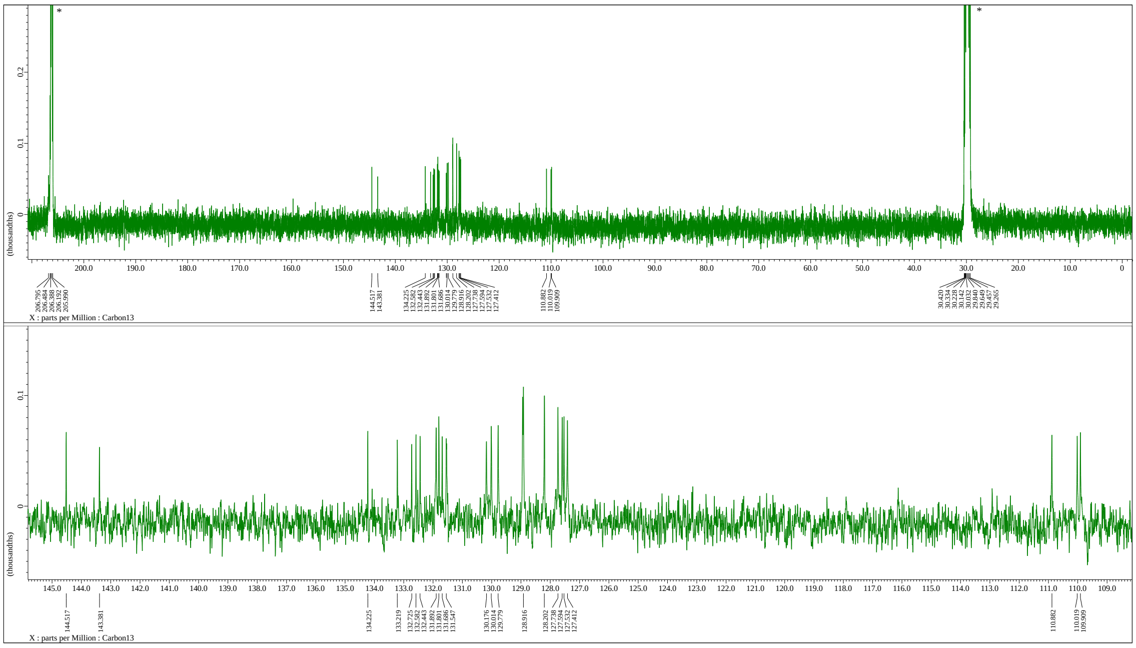

Figure S2. ^{13}C NMR spectrum of **4** in acetone-d_6 at room temperature. Peaks with * are due to the residual solvents.

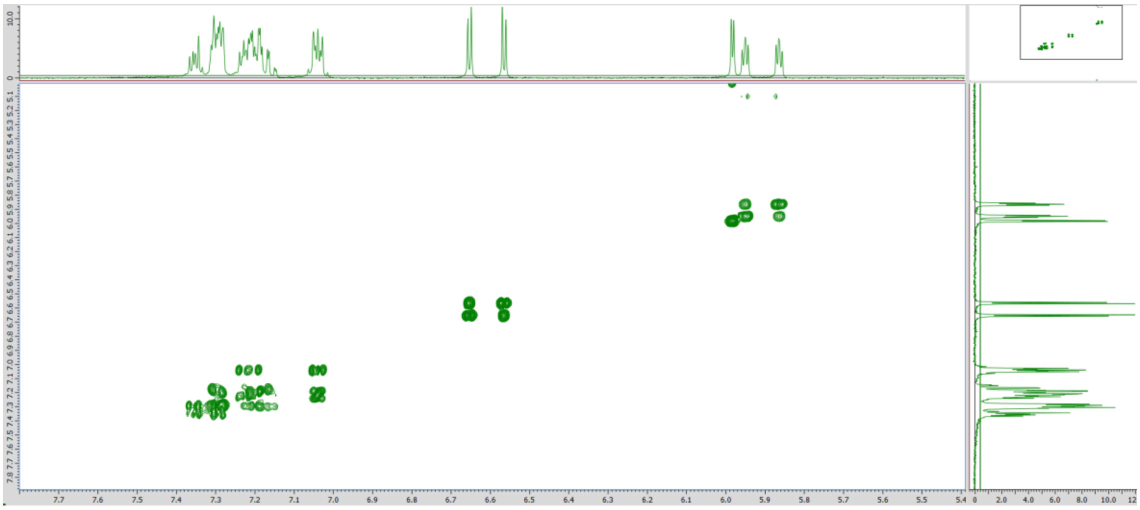

Figure S3. ^{1}H-^{1}H COSY chart of **4** in acetone-d_6 at room temperature.

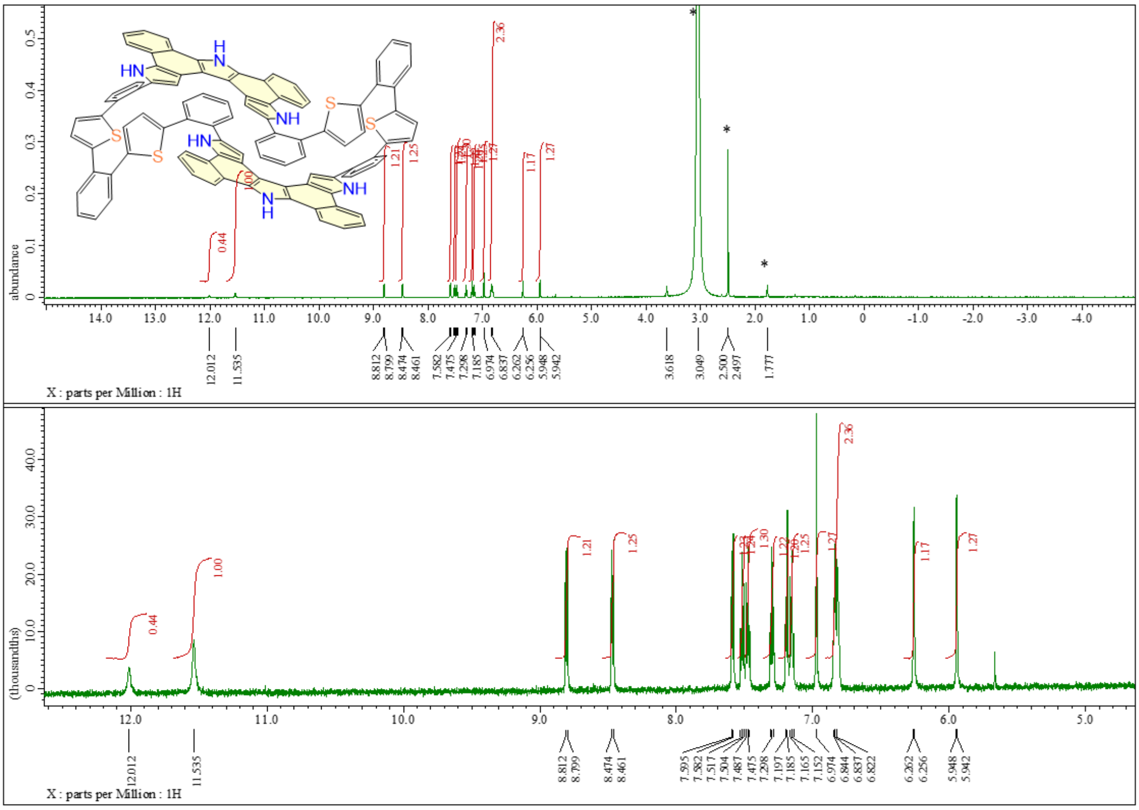

Figure S4. ^{1}H NMR spectrum of **5** in DMSO-d_6 at 100 °C. Peak with * is due to the residual solvent.

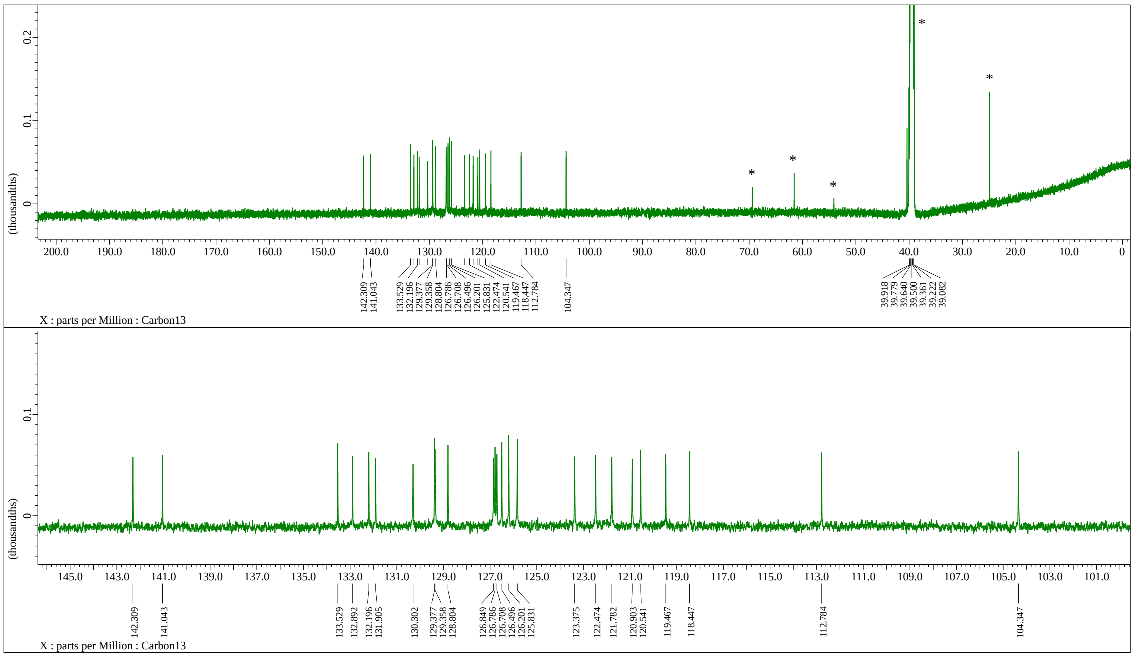

Figure S5. ^{13}C NMR spectrum of **5** in DMSO-d_6 at 100 °C. Peak with * is due to the residual solvents.

4. Mass spectra

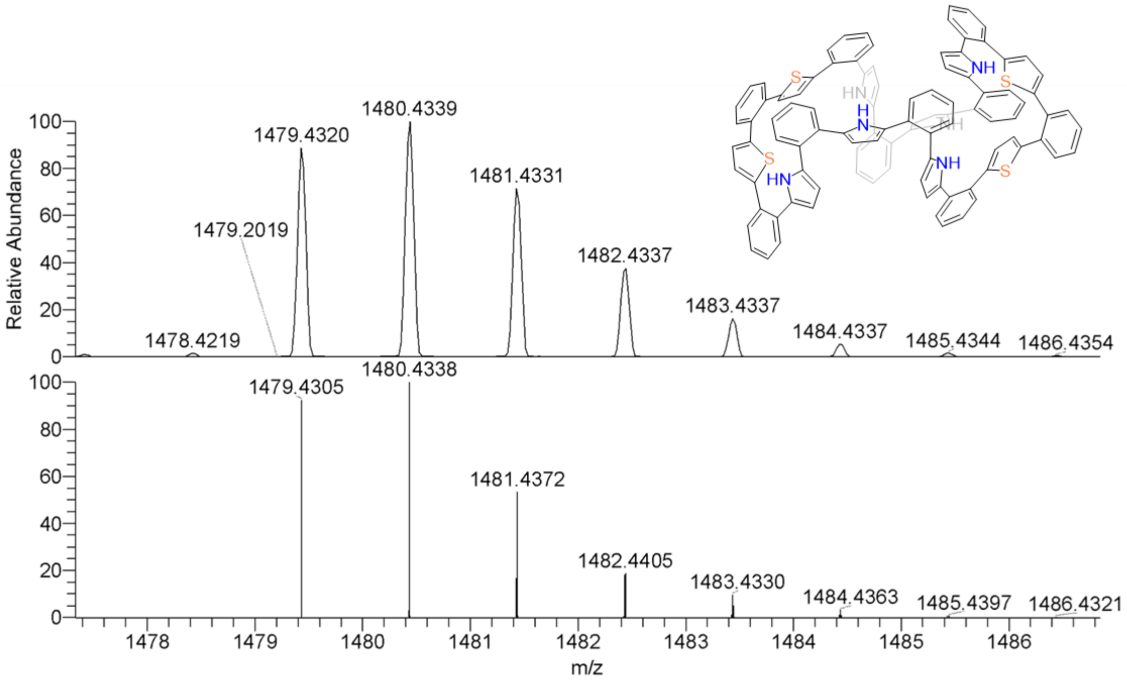

Figure S6. APCI-TOF-mass spectra of **4**. (Top; observed. Bottom; calculated [M]$^+$.)

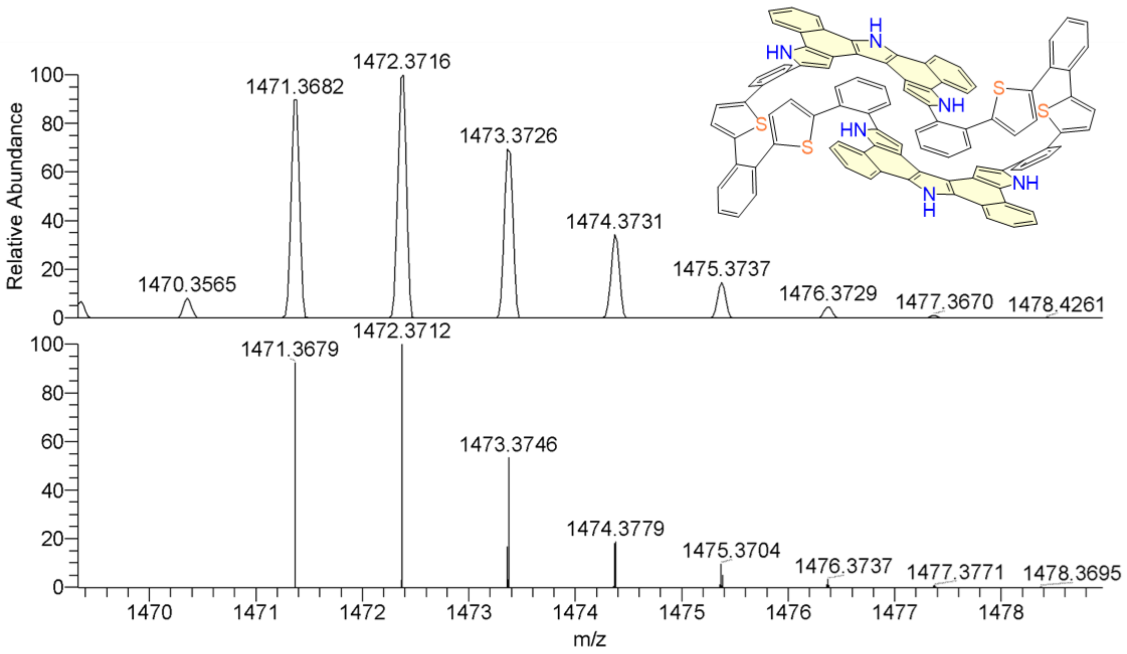

Figure S7. APCI-TOF-mass spectra of **5**. (Top; observed. Bottom; calculated [M]$^+$.)

5. X-ray crystallographic details

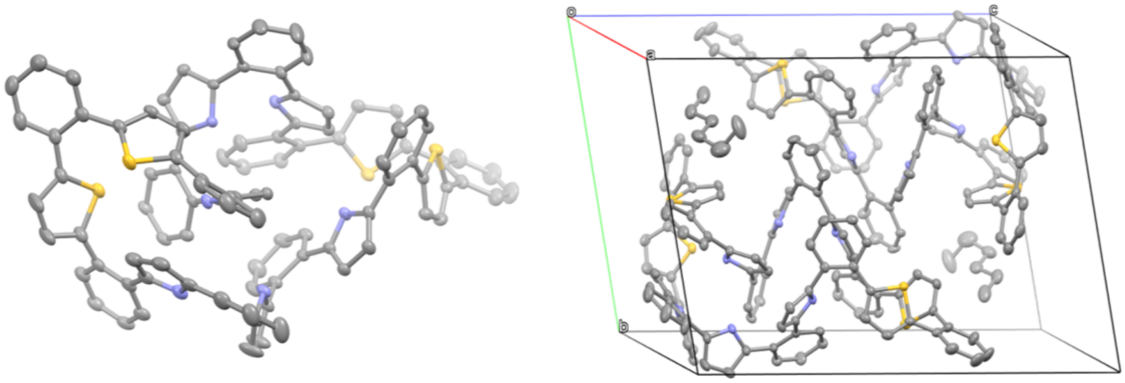

Figure S8. X-ray crystal structure of **4**. Thermal ellipsoids were scaled to 50% probability. (left) Top view and side view. (right) Packing structure. Hydrogen atoms are omitted for clarity.

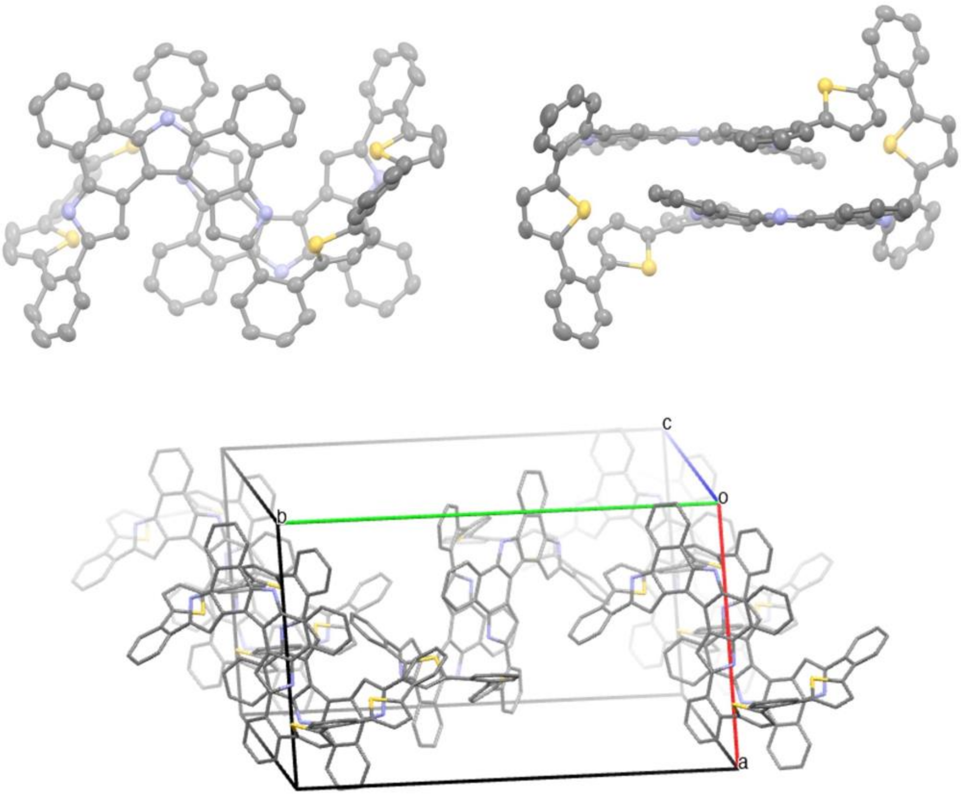

Figure S9. X-ray crystal structure of **5**. Thermal ellipsoids were scaled to 50% probability. (Top) Top view and side view. (Bottom) Packing structure. Solvent molecules (DMSO) and hydrogen atoms are omitted for clarity.

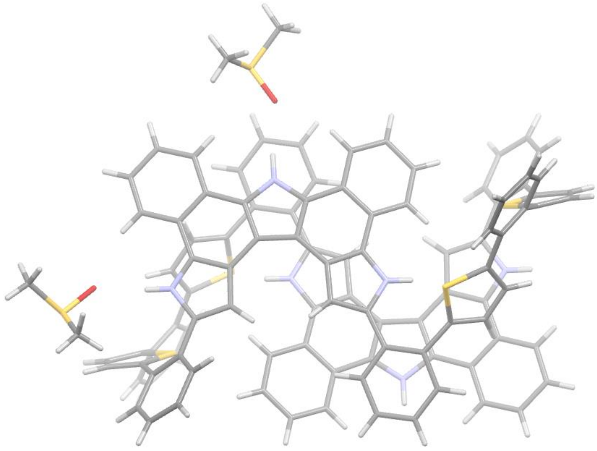

Figure S10. X-ray crystal structure of **5** with DMSO molecules coordinated to the NH sites.

Table S1. Crystal data and structure refinements for **4** and **5**.

Compound	**4**	**5**
Empirical Formula	$C_{100}H_{66}N_6S_4$, 0.666(C_6H_{14}), 0.668(C_3H_6O)	$C_{50}H_{29}N_3S_2$, 4(C_2H_6OS)
M_W	1576.01	1048.39
Crystal System	Triclinic	Monoclinic
Space Group	*P*-1 (No. 2)	$P2_1/c$ (No. 14)
a [Å]	12.9137(5)	15.0226(5)
b [Å]	15.6940(5)	25.1556(5)
c [Å]	20.8461(7)	15.0221(4)
α [deg]	80.038(3)	90
β [deg]	81.684(3)	113.837(4)
γ [deg]	85.216(3)	90
Volume [Å^3]	4110.0(3)	5192.6(3)
Z	2	4
Density [g/cm^3]	1.273	1.341
Completeness	0.968	0.996
Goodness-of-fit	1.007	1.243
R_1 [$I > 2\sigma(I)$]	0.0914	0.1008
wR_2 (all data)	0.2591	0.3095
Solvent System	acetone/*n*-hexane	DMSO/THF/H_2O
CCDC No.	2455171	2455172

6. UV–vis absorption and fluorescence spectra

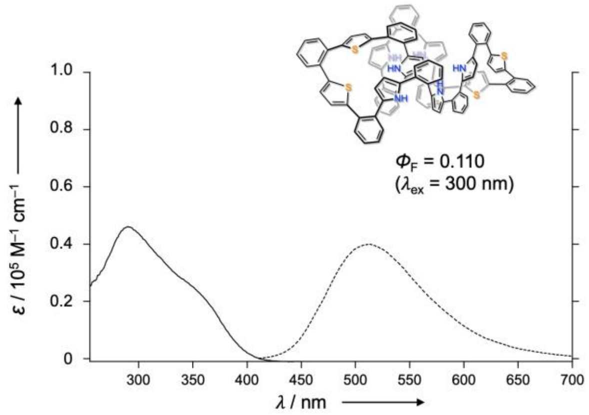

Figure S11. UV–vis absorption and fluorescence spectra of **4** in THF at room temperature.

7. DFT calculations

All calculations were carried out using the Gaussian 16 program.[S6] All structures were fully optimized without any symmetry restriction based on the structures obtained by single-crystal X-ray diffraction analysis. The calculations were performed at B3LYP-GD3BJ/def2SVP for all atoms.[S7]

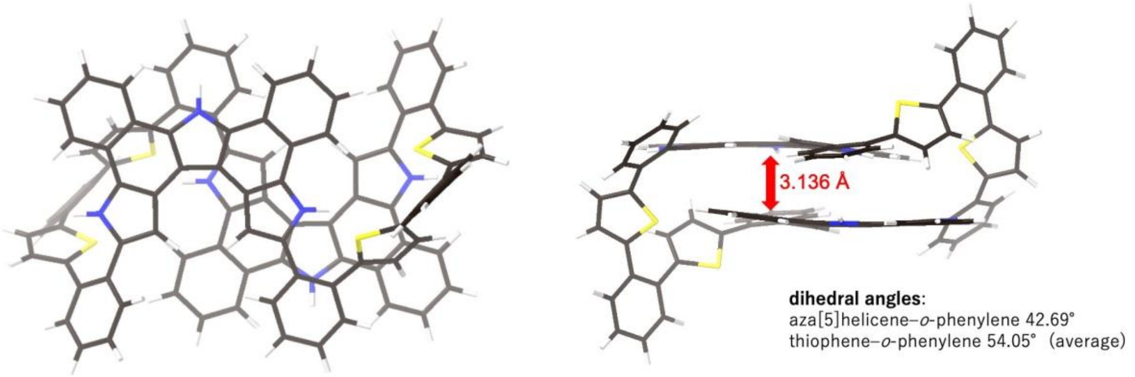

Figure S12. DFT-optimized structure of **5**.

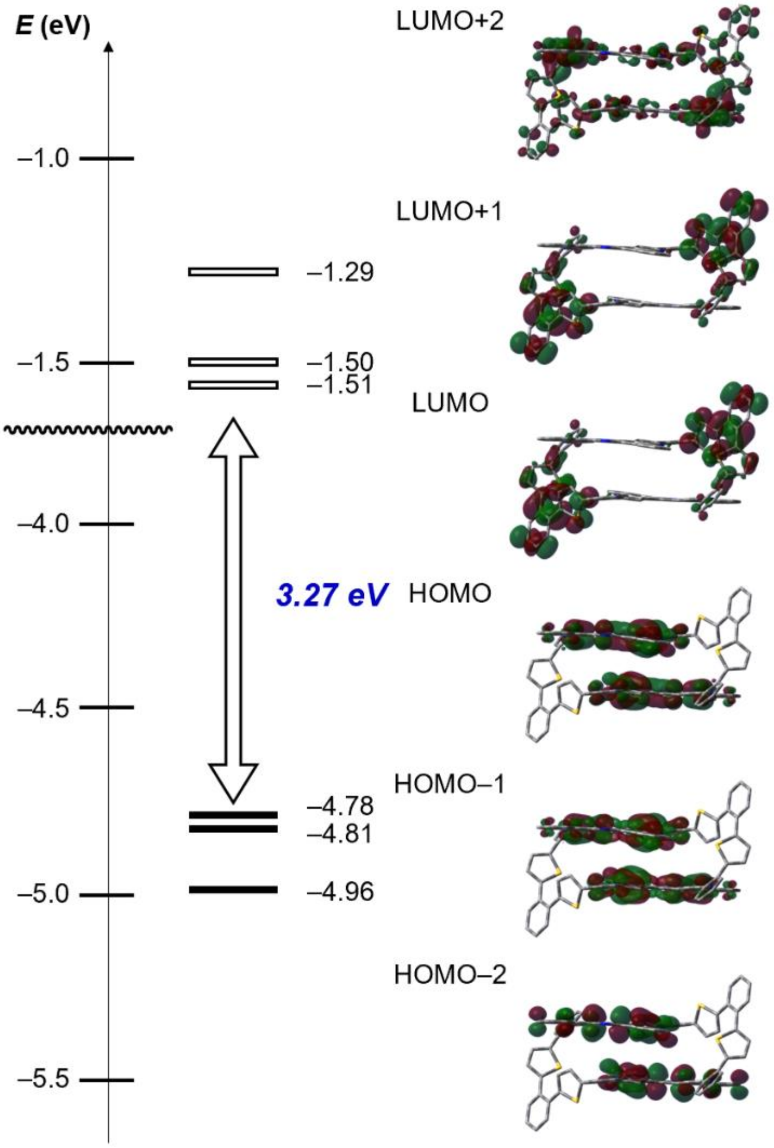

Figure S13. Kohn–Sham MO representation and energy diagrams of **5**. (Hydrogen atoms are omitted for clarity.)

Table S2. Cartesian coordinates of the optimized geometry of **5**.

Symbol	X	Y	Z
S	4.6175110	-1.9598200	2.4165150
S	6.5763980	1.3502810	0.4046300
N	-5.4330800	-0.3686700	2.6786600
H	-6.4368570	-0.3330490	2.7913780
N	-1.3725470	3.2827130	2.3655040
H	-1.3660800	4.2801600	2.2010270
N	2.1059780	-0.7033490	0.9626160
H	3.0085110	-0.7355520	0.5072490
C	-4.7287820	-1.5259170	2.4189800
C	-3.3868990	-1.1892760	2.2840510
H	-2.6033850	-1.8921320	2.0336060
C	-3.2703900	0.2223220	2.4544340
C	-4.5775310	0.7071100	2.7126920
C	-4.8959210	2.0768210	2.9598840
C	-6.2005790	2.5401710	3.2459630
H	-7.0229180	1.8239690	3.3196840
C	-6.4588490	3.8906020	3.4164790
H	-7.4749590	4.2296410	3.6298270
C	-5.4097030	4.8280990	3.3150700
H	-5.6146430	5.8924470	3.4496950
C	-4.1186670	4.4004410	3.0498590
H	-3.3154950	5.1363500	2.9756060
C	-3.8246640	3.0293930	2.8656610
C	-2.5185630	2.5380360	2.5523550
C	-2.1985700	1.1719860	2.3762920
C	-0.7942650	1.1096620	2.0629760
C	-0.3272450	2.4423880	2.0373980
C	1.0035780	2.8254890	1.6776540
C	1.4695110	4.1605030	1.7017100
H	0.8051380	4.9540050	2.0497200
C	2.7525570	4.4796290	1.2874840
H	3.0910240	5.5180960	1.3085070
C	3.6274590	3.4628560	0.8517950
H	4.6410820	3.7109050	0.5310540
C	3.2060050	2.1440970	0.8473430
H	3.8925370	1.3614780	0.5309210
C	1.8996270	1.7911030	1.2490560
C	1.4224290	0.4443650	1.2807020
C	0.1321220	0.0677910	1.7256710
C	0.0925300	-1.3526870	1.6889170
H	-0.7243620	-1.9883670	2.0031700
C	1.3200190	-1.8110650	1.2076410
C	1.7155130	-3.1925370	0.9293940
C	0.6740140	-4.1366470	0.7949460
H	-0.3529360	-3.8067840	0.9371020
C	0.9059220	-5.4515220	0.4132400
H	0.0632440	-6.1383170	0.3091420
C	2.2119230	-5.8723840	0.1389300
H	2.4160610	-6.8986300	-0.1752650
C	3.2611550	-4.9751160	0.3138960
H	4.2873250	-5.3095060	0.1579300
C	3.0515170	-3.6434140	0.7291260
C	4.2760220	-2.8452000	0.9408100
C	5.3928940	-2.8532490	0.1281540
H	5.4180360	-3.3815320	-0.8191460
C	6.4961870	-2.1383930	0.6572470
H	7.4551300	-2.0370560	0.1499000
C	6.2524500	-1.6050940	1.9054220
C	7.2114530	-0.9329950	2.7942990
C	7.2796480	-1.3173590	4.1464880
H	6.5680860	-2.0593740	4.5146640
C	8.2572410	-0.8115140	5.0003380
H	8.2880060	-1.1342370	6.0434630
C	9.2040030	0.0911640	4.5099170
H	9.9825240	0.4885280	5.1650710
C	9.1327040	0.5088370	3.1822570
H	9.8316530	1.2602130	2.8097950
C	8.1384380	0.0276470	2.3102390
C	8.0638400	0.6342560	0.9672590
C	9.0932890	0.9224610	0.0958270
H	10.1131420	0.5697210	0.2555970
C	8.6928070	1.7323760	-1.0082270
H	9.3718050	2.0797520	-1.7885720
C	7.3546940	2.0680110	-0.9860460
C	6.6398360	3.0707550	-1.8017430
C	7.2140910	4.3520550	-1.9020960
H	8.1889830	4.5220750	-1.4408080
C	6.5420910	5.4052680	-2.5192900
H	7.0086740	6.3913450	-2.5753880
C	5.2603540	5.1960930	-3.0373260
H	4.7138720	6.0165580	-3.5079830
C	4.6875860	3.9288460	-2.9728960
H	3.7084380	3.7481890	-3.4196800

C	5.3634920	2.8453620	-2.3803250
S	-4.6176710	1.9597440	-2.4163730
S	-6.5765640	-1.3500540	-0.4050430
N	5.4331500	0.3686700	-2.6787450
H	6.4369230	0.3330890	-2.7914990
N	1.3727770	-3.2828420	-2.3653210
H	1.3661630	-4.2803930	-2.2015870
N	-2.1060050	0.7030530	-0.9626570
H	-3.0085570	0.7352170	-0.5073280
C	4.7287920	1.5259360	-2.4192690
C	3.3869360	1.1892390	-2.2842400
H	2.6034040	1.8920900	-2.0338450
C	3.2704700	-0.2223840	-2.4544600
C	4.5776390	-0.7071460	-2.7126590
C	4.8960780	-2.0768670	-2.9597400
C	6.2007530	-2.5401800	-3.2457890
H	7.0230290	-1.8239280	-3.3197070
C	6.4591220	-3.8906300	-3.4160370
H	7.4752480	-4.2296380	-3.6293550
C	5.4100570	-4.8281830	-3.3143540
H	5.6150780	-5.8925530	-3.4486830
C	4.1189920	-4.4005550	-3.0492230
H	3.3158990	-5.1365300	-2.9746610
C	3.8248840	-3.0294880	-2.8653810
C	2.5187410	-2.5381500	-2.5522410
C	2.1986720	-1.1720880	-2.3762940
C	0.7943630	-1.1098230	-2.0629920
C	0.3274230	-2.4425810	-2.0373140
C	-1.0033850	-2.8257490	-1.6775900
C	-1.4692180	-4.1607980	-1.7016120
H	-0.8047880	-4.9542710	-2.0495770
C	-2.7522500	-4.4800010	-1.2873890
H	-3.0906480	-5.5184890	-1.3083980
C	-3.6272260	-3.4632740	-0.8517340
H	-4.6408150	-3.7114030	-0.5309570
C	-3.2058760	-2.1444800	-0.8473310
H	-3.8924480	-1.3619080	-0.5308930
C	-1.8995140	-1.7914070	-1.2490410
C	-1.4223770	-0.4446440	-1.2806680
C	-0.1320720	-0.0680100	-1.7256280
C	-0.0925260	1.3524480	-1.6888100
H	0.7244120	1.9881410	-2.0029000
C	-1.3200450	1.8108000	-1.2075740
C	-1.7155230	3.1922520	-0.9292350
C	-0.6740250	4.1363790	-0.7948220
H	0.3529220	3.8065400	-0.9370670
C	-0.9058990	5.4512420	-0.4130570
H	-0.0632290	6.1380560	-0.3090480
C	-2.2118850	5.8720730	-0.1386080
H	-2.4160250	6.8982880	0.1756910
C	-3.2611120	4.9748040	-0.3135430
H	-4.2872550	5.3092350	-0.1575310
C	-3.0515290	3.6431200	-0.7288680
C	-4.2760510	2.8450110	-0.9406140
C	-5.3929970	2.8532550	-0.1280330
H	-5.4181100	3.3814280	0.8193280
C	-6.4963930	2.1386460	-0.6571970
H	-7.4553850	2.0374840	-0.1499120
C	-6.2526870	1.6053430	-1.9053750
C	-7.2117400	0.9334470	-2.7943410
C	-7.2799930	1.3179770	-4.1464660
H	-6.5684090	2.0600000	-4.5145940
C	-8.2576700	0.8122590	-5.0003120
H	-8.2884940	1.1350750	-6.0434060
C	-9.2044400	-0.0904230	-4.5099320
H	-9.9830390	-0.4876430	-5.1650810
C	-9.1330870	-0.5082540	-3.1823140
H	-9.8320650	-1.2596240	-2.8098860
C	-8.1387540	-0.0272050	-2.3103230
C	-8.0640300	-0.6338920	-0.9673860
C	-9.0933570	-0.9219360	-0.0957590
H	-10.1131900	-0.5690480	-0.2553360
C	-8.6927450	-1.7318990	1.0082250
H	-9.3716300	-2.0792440	1.7886880
C	-7.3546800	-2.0676900	0.9857870
C	-6.6398840	-3.0706270	1.8013290
C	-7.2141280	-4.3519320	1.9014340
H	-8.1890210	-4.5218550	1.4401090
C	-6.5421280	-5.4052370	2.5184760
H	-7.0086740	-6.3913420	2.5743790
C	-5.2604250	-5.1961370	3.0366010
H	-4.7139840	-6.0166940	3.5071460
C	-4.6876350	-3.9288840	2.9723890
H	-3.7084950	-3.7482970	3.4192140
C	-5.3635330	-2.8453220	2.3799630

10. References

[S1] F. Chen, J. Kim, Y. Matsuo, Y. Hong, D. Kim, T. Tanaka, A. Osuka, *Asian. J. Org. Chem.*, **2019**, *8*, 994.

[S2] Y. Matsuo, K. Kise, Y. Morimoto, A. Osuka, T. Tanaka, *Angew. Chem., Int. Ed.*, **2022**, *61*, e202116789.

[S3] a) G. M. Sheldrick, *Acta Cryst. A,* **2008**, *64*, 112; b) G. M. Sheldrick, *Acta Cryst. C,* **2015**, *71*, 3.

[S4] Y. Matsuo, T. Tanaka, A. Osuka, *Chem. Eur. J.* **2020**, *26*, 8144.

[S5] Y. Matsuo, A. Osuka, T. Tanaka, *Synthesis* **2022**, *51*, 147.

[S6] Gaussian 16, Revision C.01, M. J. Frisch, G. W. Trucks, H. B. Schlegel, G. E. Scuseria, M. A. Robb, J. R. Cheeseman, G. Scalmani, V. Barone, G. A. Petersson, H. Nakatsuji, X. Li, M. Caricato, A. V. Marenich, J. Bloino, B. G. Janesko, R. Gomperts, B. Mennucci, H. P. Hratchian, J. V. Ortiz, A. F. Izmaylov, J. L. Sonnenberg, D. Williams-Young, F. Ding, F. Lipparini, F. Egidi, J. Goings, B. Peng, A. Petrone, T. Henderson, D. Ranasinghe, V. G. Zakrzewski, J. Gao, N. Rega, G. Zheng, W. Liang, M. Hada, M. Ehara, K. Toyota, R. Fukuda, J. Hasegawa, M. Ishida, T. Nakajima, Y. Honda, O. Kitao, H. Nakai, T. Vreven, K. Throssell, J. A. Montgomery, Jr., J. E. Peralta, F. Ogliaro, M. J. Bearpark, J. J. Heyd, E. N. Brothers, K. N. Kudin, V. N. Staroverov, T. A. Keith, R. Kobayashi, J. Normand, K. Raghavachari, A. P. Rendell, J. C. Burant, S. S. Iyengar, J. Tomasi, M. Cossi, J. M. Millam, M. Klene, C. Adamo, R. Cammi, J. W. Ochterski, R. L. Martin, K. Morokuma, O. Farkas, J. B. Foresman, and D. J. Fox, Gaussian, Inc., Wallingford CT, 2016.

[S7] a) A. D. Becke, *J. Chem. Phys.,* **1993**, *98*, 1372; b) C. Lee, W. Yang, R. G. Parr, *Phys. Rev. B,* **1988**, *37*, 785.
